# A Generic Control Architecture for Hybrid Micro-Machines

**DOI:** 10.3390/mi9060305

**Published:** 2018-06-19

**Authors:** Xichun Luo, Wenbin Zhong, Wenlong Chang

**Affiliations:** Centre for Precision Manufacturing, Design, Manufacture & Engineering Management (DMEM), University of Strathclyde, Glasgow G1 1XJ, UK; wenbin.zhong@strath.ac.uk (W.Z.); wenlong.chang@strath.ac.uk (W.C.)

**Keywords:** hybrid micro-machine, control architecture, system integration, software component, computer numerical control (CNC)

## Abstract

Hybrid micro-machining, which integrates several micro-manufacturing processes on one platform, has emerged as a solution to utilize the so-called “1 + 1 = 3” effect to tackle the manufacturing challenges for high value-added 3D micro-products. Hybrid micro-machines tend to integrate multiple functional modules from different vendors for the best value and performance. However, the lack of plug-and-play solutions leads to tremendous difficulty in system integration. This paper proposes a novel three-layer control architecture for the first time for the system integration of hybrid micro-machines. The interaction of hardware is encapsulated into software components, while the data flow among different components is standardized. The proposed control architecture enhances the flexibility of the computer numerical control (CNC) system to accommodate a broad range of functional modules. The component design also improves the scalability and maintainability of the whole system. The effectiveness of the proposed control architecture has been successfully verified through the integration of a six-axis hybrid micro-machine. Thus, it provides invaluable guidelines for the development of next-generation CNC systems for hybrid micro-machines.

## 1. Introduction

High value-added 3D micro-products such as optics, molds/dies, biomedical implants, and so forth are increasingly in demand. These products are usually made of a wide range of engineering materials and possess complex freeform surfaces with tight tolerance on form accuracy and surface finish. Although conventional stand-alone micro-manufacturing processes, such as micro-milling, laser machining, electrical discharge machining (EDM), and so forth, have been the major approaches in manufacturing the aforementioned products, the predictability, producibility, and productivity remain big issues [1]. In recent years, hybrid micro-machining technology has been developed to integrate several micro-manufacturing processes on one platform to tackle these manufacturing challenges, as it can achieve the so-called “1 + 1 = 3” effect, which means that the advantages of the hybrid process are more than double the advantages of the single processes [2]. The benefits of the hybrid process include the following [3]: (1) It can improve the machinability of the difficult-to-machine materials, such as ceramics, hardened steel, super alloys, and so forth; (2) It eliminates the re-alignment errors and the set-up time if the workpiece should go through sequential processes on different machines. Therefore, it provides new possibilities for high-efficiency and high-accuracy machining of some materials.

Hybrid micro-machines are distinct from conventional machines, as they accommodate more than one process on the machine, and the combination of processes varies significantly with the requirement of the application. Chavoshi and Luo [4] have classified hybrid processes into assisted and combined hybrid processes. For example, the ultrasonic-assisted diamond turning process was developed to reduce the severe tool wear when machining steel parts [5]. Laser-assisted micro-milling was developed to produce freeform surfaces on hard-brittle materials [6]. Additive manufacturing (AM) plus conventional subtractive manufacturing is a very popular configuration of the combined hybrid process. AM has the unique advantage in building complex structures [7], while the milling process can be performed afterward to obtain high dimensional accuracy and good surface finish.

To achieve the desired hybrid process, a wide range of functional modules should be integrated, such as EDM, laser, ultrasonic actuator, and so forth. Moreover, there is a growing trend for the hybrid micro-machines to integrate more functional modules, in addition to the modules for the add-on processes, for even higher efficiency and accuracy, such as the in-line metrology system for in-process surface measurement and material handling system for efficient handling of such miniature 3D micro-products. Machine developers tend to choose those modules from different vendors to satisfy the performance and cost requirements. However, those modules often possess proprietary hardware and software interfaces and the lack of plug-and-play solutions lead to tremendous difficulty in system integration, which will result in complex and inflexible CNC system, as well as a high developing cost and long lead time. The integration includes not only the hardware connection but also the intelligent coordination of different modules. The Open Architecture Control (OAC) concept was created as a potential solution to the demand of control flexibility back to 1990s, and has been promoted by many global consortiums, such as the OSE (Open System Environment for Manufacturing) in Japan [8], the OMAC (Open Modular Architecture Controllers) in the USA [9], and the OSACA (Open System Architecture for Controls within Automation Systems) in Europe [10]. Pritschow et al. [11] have summarized the criteria to assess the openness of a CNC system, the PC-based solutions with a homogenous and standardized environment were favored. Despite the significant amount of research that had been conducted, the OAC remained at the laboratory level, and had little impact in the industry; the interest in further development of the OAC had declined [12].

Modern CNC systems such as Delta Tau Power PMAC [13] and SIEMENS SINUMERIK 840D sl [14] have a certain degree of openness, but the openness is restricted to the non-kernel part of the CNC system. For example, the human-machine interface (HMI) customization with the given CNC application programming interfaces (APIs). As concluded by Pritschow et al. [11], PC-based solutions will be the future of CNC systems for hybrid micro-machines, as the PC has unprecedented I/O capabilities and ever-increasing computational speed to handle data from different functional modules with various communication interfaces. It allows for the easy incorporation of new hardware, as well as the adaption of software for different data protocols. However, due to the countless possibilities of configurations for hybrid micro-machines, a one-size-fits-all solution for the coordination of those modules to achieve the desired hybrid process is impossible. High standard expertise and a large amount of time are necessary for the seamless integration. Therefore, a generic control architecture for hybrid micro-machines will be invaluable to accelerate the integration.

The aforementioned OAC consortiums have presented some open control architectures [9,15] toward the accommodation of a broad range of functional modules. However, they require all the involved vendors to follow the standards to provide the vendor-neutral software components. Unfortunately, these guidelines have not been followed in the industry yet. In recent years, a lot of attention has been paid to the reconfigurable manufacturing system (RMS). Various architectures and frameworks have been developed [16,17] to enhance the flexibility of RMS for the increasing customization requirement in products. Those architectures and frameworks tend to take the advantage of the information and communication technology (ICT), as well as the cyber-physical system (CPS) [18,19]. However, those architectures and frameworks are at the factory level [20], while the control architecture for CNC machines is at the machine level, which requires much higher real-time performance.

This paper will present a novel generic control architecture for hybrid micro-machines, which can enhance the flexibility of CNC systems to accommodate various functional modules to achieve the desired hybrid process. The rest of the paper is organized as follows: a detailed description of the proposed control architecture is given in Section 2; the component-based technology is discussed in Section 3; the validation of the proposed control architecture is carried out in Section 4; the conclusions are drawn in Section 5.

## 2. Novel Control Architecture

**Figure 1:**
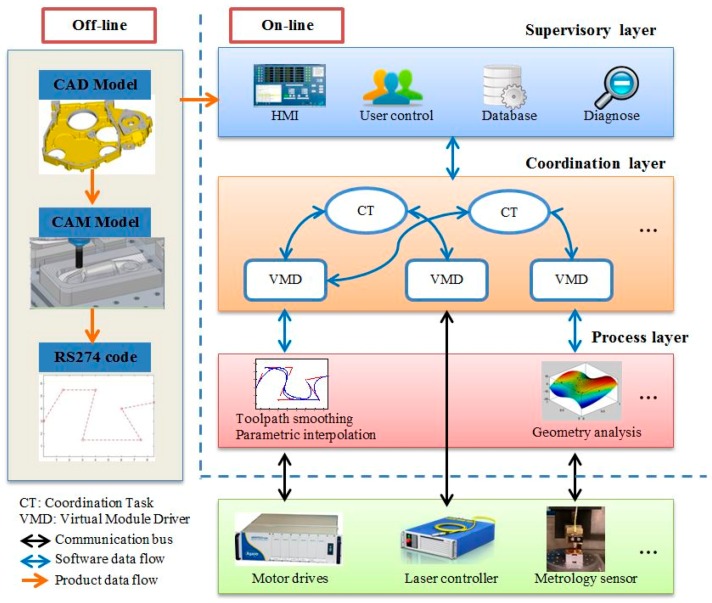
The proposed control architecture for hybrid micro-machines.

The novel generic control architecture for hybrid micro-machines proposed in this paper is divided into three layers, as shown in Figure 1. Please note that the functional modules presented in this figure represent an example of a hybrid milling/laser machine. The functions in the three layers are implemented on PC for on-line control. The off-line product data is transferred to the production via the supervisory layer, while the coordination layer synchronizes all the modules on the machine, and the process layer deals with the low-level control algorithms. The purpose of this design is to decouple the software from the hardware so that the change of hardware modules will have minimum impact on the whole system. The essential functions at each layer are encapsulated into individual software components. Each software component maintains the specific interface. Thus, the data flow between components is standardized. A loosely-coupled tightly-integrated CNC system can be achieved by implementing this architecture.

### 2.1. Supervisory Layer

The supervisory layer consists of four software components, that is, HMI, user control, database, and diagnose. The HMI provides interfaces for users to interact with the machine directly, the RS274 machining code generated from the product data can be downloaded to the interpolator through the HMI. The user control component divides machine users into two categories, that is, administrator and guest, which have different access rights to change the machine configurations stored in the built-in database. The configurations will be used to initialize the software components at the coordination layer. Data exchange between the supervisory layer and the coordination layer is performed through predefined APIs which are hardware independent. Data are collected periodically for on-line inspection and diagnosis.

### 2.2. Coordination Layer

The coordination layer performs complex data regulation in order to coordinate all the functional modules to achieve the desired hybrid process. The virtual module driver (VMD) is innovatively proposed, it is a software component that can interpret the data of a specific functional module. Therefore, the device-specific read/write operations are converted to generic read/write operations. This abstraction encapsulates the differences in data protocol and communication interface used by different vendors. Therefore, the consistent interaction with a functional module is achieved. For example, the VMD maintains the following laser manipulation interfaces for any laser controllers, while the implementation of those methods is device-specific:

- Enable or disable the laser controller.
- Start or stop the laser emission.
- Set the laser output power.
- Set the laser output frequency.

The coordination task (CT) is the software component developed for the data exchange and synchronization between specified functional modules. Because the data has been interpreted in the VMD, the CT can concentrate on the synchronization logic, which is hardware independent. The coordination tasks should be executed in the real-time environment or in the normal desktop environment with a high priority to guarantee the highest data throughput and the lowest delay. The process specified adaptive control algorithms can also be implemented in the CT, for example, laser power adjustment after analyzing the surface results from the metrology module.

The coordination layer makes the proposed control architecture fundamentally different from the conventional control architectures, which only regulates a single process. It can enhance the system’s flexibility enormously as discussed in the following cases:

1. When a functional module is upgraded, only the corresponding VMD component should be adapted for the new data protocol or communication bus, but maintains the original software interface. The whole system can work seamlessly without modifying any other software components.
2. If a new functional module is to be added, a corresponding VMD component should be developed. If it needs to interact with other functional modules, a new CT should be developed by invoking the given interfaces provided by the related VMDs. The other part of the system will not be affected.

### 2.3. Process Layer

**Figure 2:**
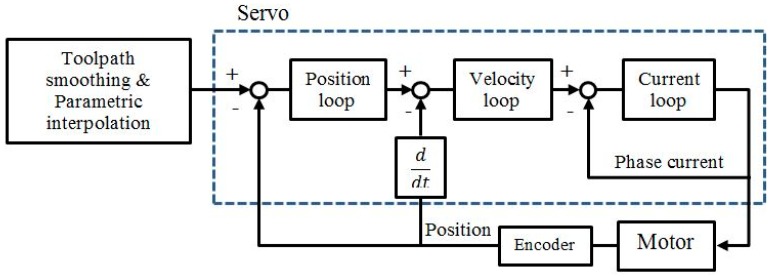
The motion control module structure.

The process layer executes intelligent algorithms in real-time for the related functional modules. Motion control module is one of the basic modules for a hybrid micro-machine. It mainly consists of two parts, that is, the interpolator and the servo, as shown in Figure 2. The interpolator can interpret the RS274 code and convert the segmented toolpath to parametric curves to avoid the feed-rate and acceleration fluctuation problem caused by the linear interpolator [21]. Then, the built-in parametric interpolator generates reference positions for the servo system. The servo system generally consists of the three cascaded control loops in order to follow the position commands. The communication between the interpolator and the servo is usually achieved by high-speed deterministic buses, such as EtherCAT developed by Beckhoff Automation, and FireWire used by Aerotech. This design allows distributed hardware control. The servo part can be closer to the actual motor and feedback sensors.

Sensor data processing algorithms are executed in this layer as well, such as the workpiece surface analysis. Those functional modules that do not require intelligent algorithms are connected directly to the coordination layer. For example, for the laser process, some simple laser manipulation logics are enough.

## 3. Component-Based Technology

**Figure 3:**
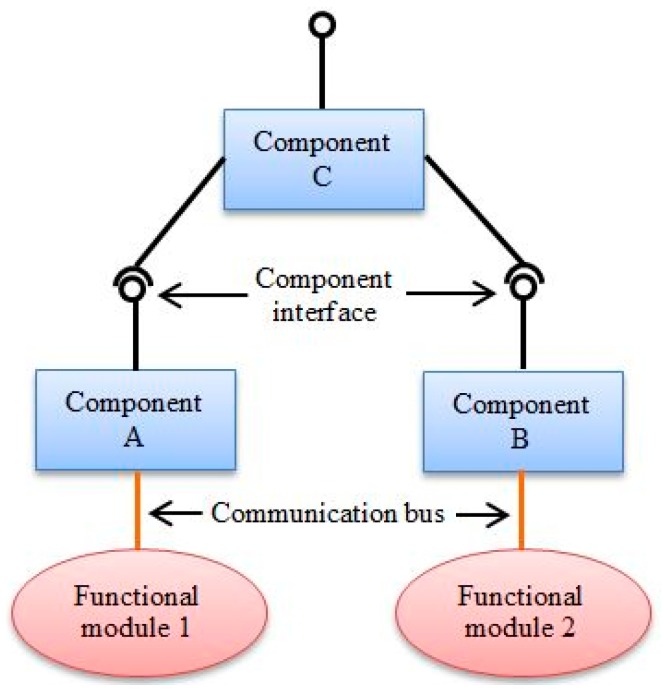
An example of the component-based system.

A number of software components are used to encapsulate the functions at each layer of the proposed control architecture, which can be used as building blocks for the structured development of the control architecture. Individual software components communicate with each other through interfaces. The interface defines a set of actions which are understood by both the original component and the target component [22]. Figure 3 illustrates an example of the component-based system. Component A and B provide the necessary abstraction for the function modules, that is, convert device-specific write/read operations to generic write/read operations. Component C encapsulates the coordination algorithm for the two function modules, it calls the interfaces provided by Component A and B to finish the hardware manipulation. Component C exposes an interface for the other components to configure it as well.

Building systems from components has seen wide applications in the industry control domain. For example, many component-based software frameworks have been created for robotics systems to support the development and reuse of “large grained” pieces of robotics software [23]. The AUTomotive Open System ARchitecture (AUTOSAR) [24] maintains standardized architecture and architecture components for automotive applications. The Open Platform Communications (OPC) Foundation [25] has developed and maintained a specification that defines standard interfaces for process automation with the component-based technology. The major benefits to use software components include the following:

- Software components are reusable. Component provides service to the system via its interface, the data structure and algorithms are encapsulated. If two components have the compatible provided interface and required interface, they can replace each other without breaking the system. The development of the system is, therefore, accelerated.
- The system scalability and flexibility are increased. Components are treated as the building blocks of the CNC system. Various control architectures can be constructed by combining the necessary components.
- The system maintainability is improved. An upgraded component can replace the old component, while the other components in the system are not affected. The component is linked dynamically at run-time. There is no need to re-compile other components.

Software components are composed by component models. Many component models have been proposed for different applications. The proposed control architecture will be implemented on the Microsoft Windows desktop environment in this paper. Microsoft has deemphasized the component object model (COM) for .NET. As a result, the software components for the control architecture will be developed with .NET technology. The C#, Visual Basic, and C++/CLI programming languages can be selected. The software components are implemented with classes in dynamic link libraries (DLLs) or in executable files.

## 4. System Integration of a Six-Axis Hybrid Micro-Machine

### 4.1. Overview of the System Integration

**Figure 4:**
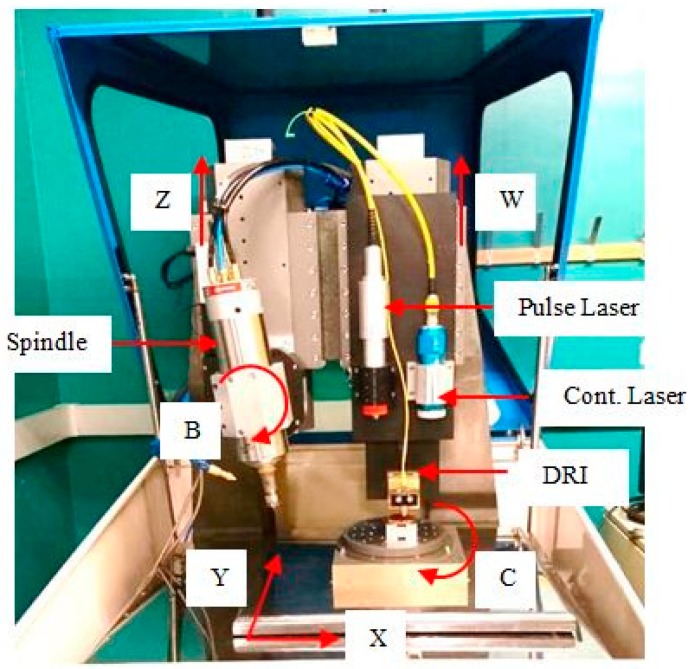
The configuration of the six-axis hybrid micro-machine.

**Figure 5:**
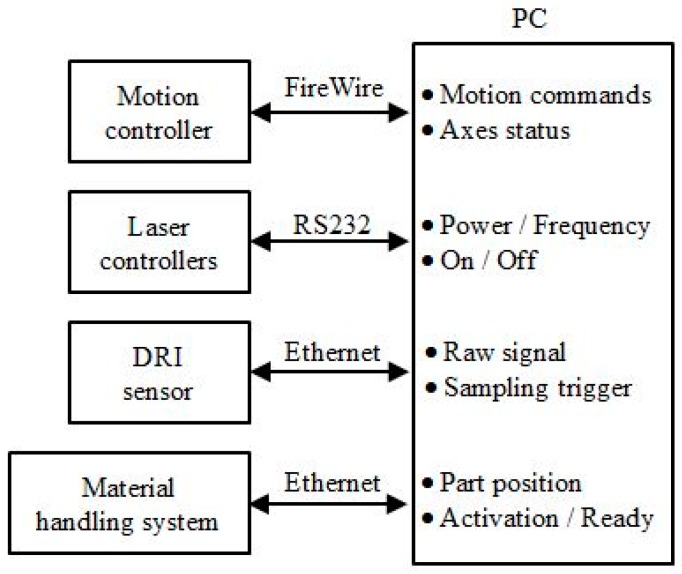
The communication of the functional modules.

The proposed control architecture is applied in the integration of a six-axis hybrid micro-machine, as shown in Figure 4. The machine is capable of laser machining, laser-assisted micro-milling and laser-assisted micro-grinding operations. It incorporates a nanosecond pulse laser, a high-power continuous laser, a dispersed reference interferometry (DRI) sensor and a material handling system. The design of the machine is presented in Reference [26]. The motion controller is connected to a PC via a high-speed deterministic FireWire bus. It receives motion commands from the PC and feedbacks axes status. The two laser controllers are connected using the RS232 bus. Data are transmitted on the bus include laser power and frequency parameters, as well as laser on/off commands. Both the DRI and material handling system are connected using the Ethernet bus, which can provide high bandwidth for the sensor raw data and other communication data. The simplified communication scheme of the system is shown in Figure 5.

### 4.2. Integration of Motion Controller

The motion controller is in charge of the movement of the six axes and the spindle. The linear axes (*X*, *Y*, *Z*, *W*) are driven by linear motors and supported by micro recessed air bearings, so that the friction and backlash can be eliminated, which leads to better positioning accuracy and higher attainable acceleration. Easy maintenance is also achieved as no lubrication is required. Each linear axis uses a linear encoder with 20 μm pitch as the position and velocity feedback sensor for the servo system. The feedback resolution can achieve nanometer level with up to 65536 encoder multiplier. Workpieces are fixed on the rotary C table, which is mounted on the top of the stacked *X Y* axes. The C axis is driven by a brushless DC motor with air bearing. A rotary encoder with 15,744 lines is used as the feedback sensor. The rotary B axis is mounted on the vertical *Z* axis. It is driven by the worm-gear mechanism, which offers high structural stiffness and long-term stability for the spindle that is fixed on the *B* axis. The heavy-duty spindle can reach up to 50,000 rpm with liquid cooling.

**Figure 6:**
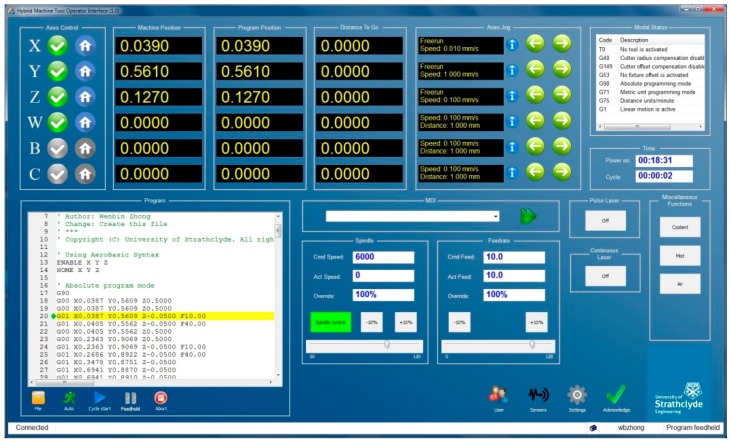
The customized HMI component.

The Aerotech A3200 motion controller is chosen as the motion control module. The A3200 interpolator is implemented in the INtime^®^ for the Windows real-time kernel. The synchronized motion is achieved by sending the reference positions to the drive of each axis with the high-speed deterministic FireWire bus. However, the A3200 is not open for users to implement customized interpolation algorithms at the process layer. The VMD for the A3200 controller is provided by Aerotech in the form of .NET components. Many classes are provided to interact with the controller. Through the interfaces of the VMD, the HMI module can write motion commands to and acquire axes information from the controller easily, as shown in Figure 6.

### 4.3. Integration of Laser Controllers

**Figure 7:**
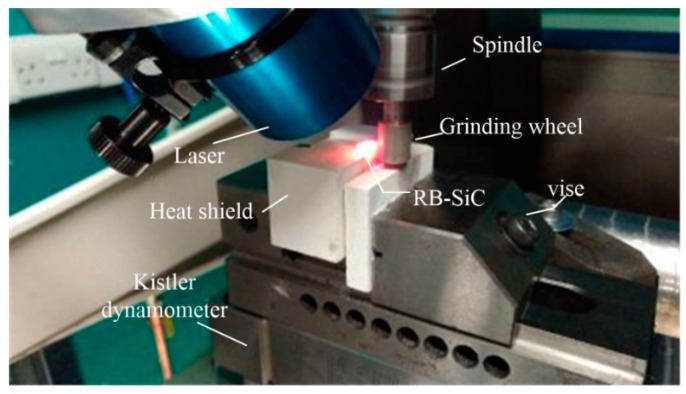
The laser-assisted micro-grinding process.

Two laser systems are integrated on the hybrid micro-machine. A nanosecond pulse laser is used to manufacture microstructures or remove micro burrs that are generated by the micro-milling process. The maximum power of the pulse laser is 20 W and the spot size is 15 μm in diameter. The continuous laser is used to assist the micro-milling and micro-grinding process by preheating the workpiece, as shown in Figure 7. The maximum power of the continuous laser is 200 W, and the spot size is 4 mm in diameter.

**Figure 8:**
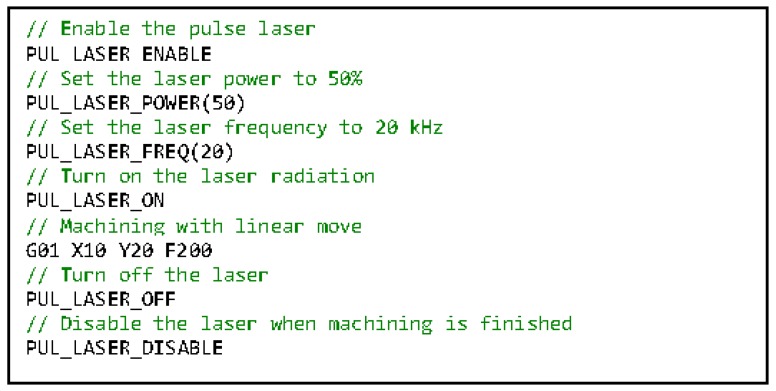
An example of the laser hybrid machining code.

Both laser controllers use RS232 to communicate with the PC. The laser controllers are connected to the coordination layer in the proposed control architecture, as no real-time algorithms are required to process their data. Although the laser controllers use identical communication buses, the data protocols are different. Two VMD components should be developed separately to convert the generic laser operations to the device-specific operations. The best practice to achieve the aforementioned laser assisted hybrid process is to embed the laser control logic in the RS274 program so that the users have the flexibility to manipulate the laser at any location of the program to satisfy the requirement of the hybrid process. However, it is impossible for the interpolator to use the laser VMD interface directly from the real-time environment, which is separated from the normal desktop environment. Therefore, a coordination task should be developed to synchronize interpolator commands and actual laser operations. For the interpolator, all the laser control logic is implemented as preprocessors or macros, which are similar to the conventional M codes in the RS274 standard. An example of the laser hybrid machining code is shown in Figure 8. Each preprocessor or macro actually changes the value of a particular interpolator global variable. The coordination task continuously reads the values of those variables through the interpolator VMD. If a value change is monitored, the coordination task will invoke the corresponding laser VMD interface method to finish the laser manipulation.

### 4.4. Integration of DRI in-Line Metrology System

**Figure 9:**
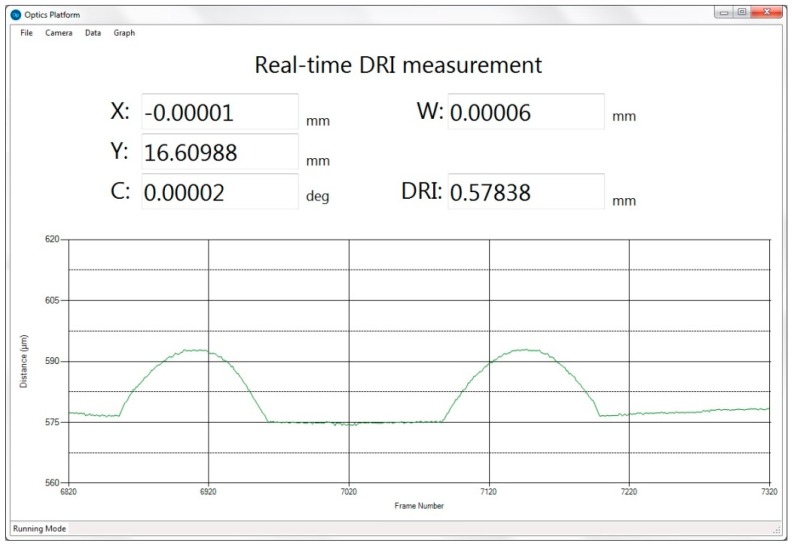
The DRI measurement coordination task.

The DRI is a single point variant of white light interferometry. It can achieve in-line non-contact metrology. The vertical resolution of DRI is 5 nm, the measurement range is 600 μm. The raw interferogram signal is transmitted to the PC using Ethernet. Complex signal processing algorithms have been implemented in an executable component. The DRI signal processing component only gives the current distance from the probe to the workpiece surface. The measurement height and the workpiece position should be synchronized to achieve the 3D position. A surface profile is achieved with many equally spaced scanned positions along a particular scanning direction. The surface topography is composed of many profiles. Figure 9 shows the measured surface profile as a result of the coordination task through reading the workpiece position and the DRI measurement height simultaneously via their VMD interfaces.

### 4.5. Integration of a Material Handling System

**Figure 10:**
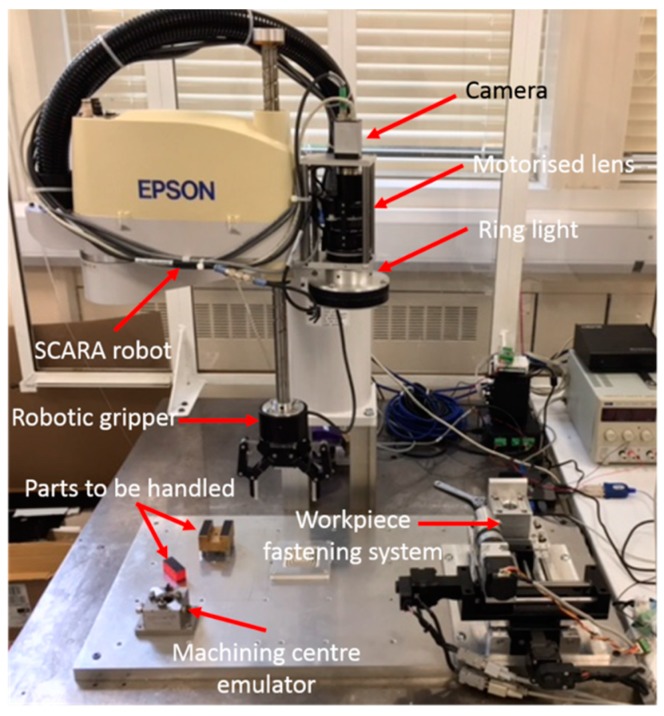
The material handling system.

A complete material handling system, consisting of a SCARA (Selective Compliance Assembly Robot Arm) robot, a two finger gripper, and a vision system, as shown in Figure 10, is also required to be integrated on this hybrid micro-machine. The maximum reach of the material handling system is 650 mm with a repeatability of 30 μm. The maximum payload is 5 kg and the gripping force range is 5–50 N. To avoid the high-degree interconnection between the material handling system and the hybrid micro-machine, as well as to simplify the reconfiguration of the hardware platforms, the controllers of the material handling system are integrated on a stand-alone PC. The communication between the two control systems is via Ethernet.

The hybrid machine needs to send the position of the part to the material handling system, which has a standalone motion controller. Then the material handling system will load or unload the part automatically. When the loading or unloading is completed, the completion signal is sent to the hybrid machine. A typical communication cycle between the two control systems includes command/data from one side and acknowledgment from the other side. Like the VMD for the laser controller, the VMD for the material handling system encapsulates the low-level Ethernet read/write operations and the predefined communication protocol. The coordination task continuously monitors the status of the A3200 interpolator and the material handling system and performs corresponding actions. For example, when the interpolator finishes running all the machining code, the coordination task will send the complete command to the material handling system to initiate the material unloading process.

## 5. Conclusions and Future Work

This paper presents a novel control architecture for hybrid micro-machines, which is made up of the following layers:

- Supervisory layer: it is in charge of the human-machine interaction and monitoring.
- Coordination layer: it provides an abstraction for each functional module and hosts the coordination tasks.
- Process layer: it executes the intelligent algorithms in real-time for related functional modules.

The essential functions at each layer are encapsulated into individual software components. The proposed control architecture can enhance the flexibility of the CNC system to accommodate a broad range of functional modules. The component design also improves the scalability and maintainability of the whole system. The effectiveness of the proposed control architecture has been successfully verified through the integration of a six-axis hybrid micro-machine. Thus, it provides invaluable guidelines for the development of next-generation CNC systems for hybrid micro-machines.

The future work of this research is to integrate the proposed control architecture in an industry 4.0 system. The PC can provide convenient hardware cyber connection to the higher level system. The challenge remains in the development of additional software components at the supervisory layer to exchange specific data with remote servers.
